# Association of HOTAIR, MIR155HG, TERC, miR-155, -196a2, and -146a Genes Polymorphisms with Papillary Thyroid Cancer Susceptibility and Prognosis

**DOI:** 10.3390/cancers16030485

**Published:** 2024-01-23

**Authors:** Jelena Karajovic, Bozidar Kovacevic, Bojana Uzelac, Debora Stefik, Bojana Jovanovic, Petar Ristic, Snezana Cerovic, Gordana Supic

**Affiliations:** 1 Clinic for Endocrinology, Military Medical Academy, 11000 Belgrade, Serbia; karajovicjelena@gmail.com (J.K.); petris011@yahoo.com (P.R.); 2 Institute for Pathology and Forensic Medicine, Military Medical Academy, 11000 Belgrade, Serbia; bozociti@yahoo.com (B.K.); bojana.jovanovic.vma@gmail.com (B.J.); cerovics@gmail.com (S.C.); 3 Medical Faculty of Military Medical Academy, University of Defense, 11000 Belgrade, Serbia; 4 Institute for Medical Research, Military Medical Academy, 11000 Belgrade, Serbia; bojanicamb@gmail.com (B.U.); deborastefik@gmail.com (D.S.)

**Keywords:** papillary thyroid carcinoma, HOTAIR, MIR155HG, miR-155, polymorphisms, prognosis, cancer risk, ATA risk

## Abstract

**Simple Summary:**

Although papillary thyroid carcinoma (PTC) has a relatively indolent behavior, the clinical course in patients with recurrent or metastatic disease is still unfavorable. Polymorphisms in long non-coding RNA and microRNA genes may play a significant role in PTC. Thus, we evaluated the association of HOTAIR rs920778, MIR155HG rs1893650, TERC rs10936599, miR-155 rs767649, miR-196a2 rs11614913 and miR-146a rs2910164 polymorphisms with the PTC risk and prognosis, in 102 PTC patients and 106 controls. Our results showed that the HOTAIR rs920778 polymorphism is associated with an increased PTC risk, as well as with lymph node metastasis, recurrence, and progression-free survival. Multivariate Cox regression revealed that ATA risk and HOTAIR rs920778 polymorphism are independent prognostic factors in PTC. In addition, we observed a novel association of the MIR155HG rs1893650 polymorphism with the reduced PTC risk. Polymorphisms in HOTAIR and MIR155HG genes could potentially be new biomarkers for risk assessment and prognosis in PTC patients.

**Abstract:**

Polymorphisms in long non-coding RNA and microRNA genes may play a significant role in the susceptibility and progression of papillary thyroid carcinoma (PTC). The current study investigates the polymorphisms HOTAIR rs920778, MIR155HG rs1893650, TERC rs10936599, miR-155 rs767649, miR-196a2 rs11614913 and miR-146a rs2910164 in 102 PTC patients and 106 age- and sex-matched controls of the Caucasian Serbian population, using real-time PCR. We observed differences in genotype distributions of the HOTAIR rs920778 (*p* = 0.016) and MIR155HG rs1893650 (*p* = 0.0002) polymorphisms between PTC patients and controls. HOTAIR rs920778 was associated with increased PTC susceptibility (adjusted OR = 1.497, *p* = 0.021), with the TT variant genotype increasing the risk compared to the CC genotype (OR = 2.466, *p* = 0.012) and C allele carriers (CC + CT) (OR = 1.585, *p* = 0.006). The HOTAIR rs920778 TT genotype was associated with lymph node metastasis (*p* = 0.022), tumor recurrence (*p* = 0.016), and progression-free survival (*p* = 0.010) compared to C allele carriers. Multivariate Cox regression revealed that ATA risk (HR = 14.210, *p* = 0.000004) and HOTAIR rs920778 (HR = 2.811, *p* = 0.010) emerged as independent prognostic factors in PTC. A novel polymorphism, MIR155HG rs1893650, was negatively correlated with susceptibility to PTC, with TC heterozygotes exerting a protective effect (OR = 0.268, *p* = 0.0001). These results suggest that the polymorphisms HOTAIR rs920778 and MIR155HG rs1893650 could be potential prognostic and risk biomarkers in papillary thyroid carcinomas.

## 1. Introduction

Papillary thyroid carcinoma (PTC) is the most common type of endocrine neoplasm of the thyroid gland. Early detection, appropriate surgical treatment, and ^131^I therapy contribute to a favorable prognosis of PTC cases. However, the incidence of this malignancy has risen over the last decades, while the clinical course in patients with recurrent or metastatic disease is still unfavorable [1]. Clinicopathologic factors associated with potentially more aggressive clinical behavior of PTC include age (>55 years), male sex, stage, lymph node metastases, tumor size, multifocality, histological subtype, extrathyroidal extension, ATA (The American Thyroid Association) risk, and absence of Hashimoto’s thyroiditis [1,2,3]. Furthermore, a high number of differentially expressed genes associated with the aggressiveness of thyroid cancer were found when the age cut-off point was changed to 55 years [3].

Several recent studies indicate the impact of epigenetic changes on PTC’s development and biological behavior [4,5]. Epigenetic changes are dynamic and potentially reversible changes in gene expression that occur without changes in DNA sequence. The main mechanisms include DNA methylation, histone modifications, and noncoding RNAs that act as negative regulators of gene expression at the post-transcriptional level. Noncoding RNAs (ncRNAs) comprise a very heterogeneous family of RNA molecules, including long noncoding RNAs (lncRNAs) and microRNAs (miRNAs, miRs), which have emerged as key regulators of gene expression in numerous biological processes and malignancies, including PTC [4,5,6]. Noncoding RNAs are generally subdivided based on their length into microRNAs, the single-stranded RNAs that are about 18–22 nucleotide-long, and lncRNAs, which are substantially longer, over 200 nucleotides. In humans, approximately 2000 miRNAs have been discovered, and they cause the target messenger RNA’s degradation or translational inhibition. Each miRNA can silence the expression of over 100 genes, and a single mRNA could potentially be the target of multiple miRNAs [6]. The human genome contains around 30,000 lncRNAs, most of which are non-conserved. LncRNAs act on fewer genes with higher specificity, affecting the epigenome by interacting with transcription factors and/or chromatin-modifying complexes [5,6]. By competing for the binding of microRNAs, lncRNAs can act as competing endogenous RNAs (ceRNAs), acting as molecular sponges [7]. The complex cross-talk between various lncRNAs and miRNAs plays a role in orchestrating malignant transformation and fine-tuning gene expression to create a protumor microenvironment [8]. Although lncRNAs were originally defined as noncoding, recent studies show that several lncRNAs have the potential to encode functional micropeptides that influence immune response [9], and certain mechanisms initially attributed to lncRNAs might be affected by their micropeptides.

HOX transcript antisense RNA (HOTAIR) is a polyadenylated lncRNA that is 2.2 Kb long and includes six exons. It is transcribed from the antisense strand of the HOXC cluster on chromosome 12q13.13. Multiple studies have shown that HOTAIR is overexpressed in a variety of cancers, including PTC [6,10,11,12,13,14,15]. The oncogenic potential of HOTAIR is based on its unique ability to act as an epigenetic master regulator of chromatin dynamics that promotes transcriptional silence of target genes [14]. HOTAIR contains different binding sites for histone modification enzymes, with the 5′ domain involved in binding the Polycomb Repressive Complex 2 (PRC2) and the 3′ domain involved in binding the lysine-specific histone demethylase 1A (LSD1). HOTAIR acts as a molecular scaffold that recruits PRC2 and LSD1/CoREST/REST complexes directly into the promoter regions of multiple cancer-associated genes [16]. In addition, HOTAIR acts as a ceRNA that sponges microRNAs and reverses the repression of their targets, resulting in a complex cross-talk between overexpressed HOTAIR and various microRNAs that affect cancer cell proliferation, migration, and invasion [17].

MicroRNA-155 host gene (MIR155HG), also referred to as lncRNA-155 and B-cell integration cluster transcript (BIC), is a 1.5 Kb gene with three exons located on chromosome 21q21.2. Its exon 3 is highly conserved and encodes a precursor of miR-155, which plays a crucial role in inflammation and anti-tumor immune responses [18]. A growing body of evidence has revealed that the MIR155HG locus plays a dual role in the regulation of innate immunity by encoding the lncRNA MIR155HG in addition to processing miR-155. Aberrant expression of MIR155HG is associated with various malignancies [19,20,21]. Direct regulation of the MIR155HG promoter by nuclear factor kappa-B (NF-κB), an essential transcription factor involved in the regulation of innate immunity and inflammation, increases the levels of both MIR155HG and mature miR-155 [22]. MIR155HG also acts as a ceRNA to sponge several miRNAs, including miR-155 [20,21]. Interestingly, MIR155HG encodes a micropeptide that suppresses the inflammatory response via modulating antigen presentation [9].

The telomerase RNA component (TERC), a 451 nt long RNA molecule encoded by the 3q26.2 locus, is an essential component of telomerase. The main functions of TERC are to serve as a template for telomeric repeat addition by the catalytic telomerase reverse transcriptase (TERT) and to act as a molecular scaffold that provides binding sites for telomeric regulatory proteins [17,23]. In addition, TERC contains binding motifs that serve as a scaffold for tumor suppressors related to the NF-κB pathway and influence cellular inflammation [17,23]. In a number of TERT-expressing malignancies, TERC overexpression drives tumor progression [24], as well as in aggressive thyroid carcinomas, where its upregulation is independently associated with progression-free survival [25].

MiR-146a and miR-155, the two most thoroughly studied miRNAs, emerged as essential regulators of immunological and inflammatory signaling with opposing activities, with miR-146 acting as an anti-inflammatory and miR-155 as a proinflammatory counterpart [26]. Both miR-146a and miR-155 have dual functions and can act as a tumor suppressor or oncogene in various malignancies [18]. Changes in miR-155 and miR-146a expression have previously been associated with PTC risk and prognosis [4,27,28]. MiR-196a is another inflammation-associated microRNA whose expression is an independent prognostic factor for poor prognosis in PTC [4,29].

To our knowledge, single nucleotide polymorphisms (SNPs) in the MIR155HG and TERC, as well as miR-155 and miR-196a2 genes, have not been studied in PTC patients, while a limited number of studies examined the polymorphisms in the HOTAIR and miR-146a genes in PTC [30,31,32,33]. Thus, our study aims to investigate the association of HOTAIR rs920778, MIR155HG rs1893650, TERC rs10936599, miR-155 rs767649, miR-196a2 rs11614913 and miR-146a rs2910164 genetic variations with the cancer risk, progression, and progression-free survival of PTC patients.

## 2. Materials and Methods

### 2.1. Study Population

The cohort included 102 patients who underwent total or nearly total thyroidectomy, with or without lymphadenectomy, in the Clinic for General Surgery of the Military Medical Academy (MMA) in Belgrade, Serbia and were monitored postoperatively in the Clinic of Endocrinology, MMA. The study included patients with pathohistologically diagnosed classic subtype of PTC, both sexes, older than 18 and younger than 80 years. Patients who had undergone thyroid lobectomy, patients without follow-up data, those with other subtypes of PTC, or those with a history of cancer were excluded from the study. General clinical data such as age, sex, type of surgery, presence of local and/or distant metastasis, and disease recurrence were obtained from the medical records. The control group consisted of 106 age- and sex-matched healthy individuals who were randomly recruited at annual routine examinations in the Clinic for Endocrinology, MMA. All participants were Caucasians of Serbian origin.

### 2.2. Histopathological Analysis, Pathological Evaluation, and Tumor Staging

The pathohistological examination was carried out at the Institute of Pathology and Forensic Medicine, MMA. Pathohistological features were examined on formalin-fixed, paraffin-embedded cancer tissue sections stained using the standard hematoxylin–eosin method. Pathological evaluation and TNM staging were performed by two pathologists (BK, SC). Histological parameters were analyzed according to the 5th edition of the World Health Organization Classification of Tumors of Endocrine Organs [34]. The following pathohistological parameters were examined in each case: tumor size, histological subtype, presence of lymph node metastases, gross extrathyroidal extension (ETE), vascular invasion, tumor multifocality, stromal calcification, and coexistence of Hashimoto’s thyroiditis. The 8th edition of the AJCC/TNM Staging System for PTC was assessed for the TNM classification, and the age cut-off of 55 years was used for risk stratification in tumor staging [35]. The ATA risk stratification system was used to predict the 1-year risk of PTC relapse [2].

### 2.3. DNA Isolation and Gene Polymorphism Analysis

Genetic analysis was performed in the Department of Molecular Genetics, Institute for Medical Research, MMA. The commercial kit Extract Me, Poland, was used to extract genomic DNA from FFPE-PTC tissue samples. Real-time PCR with commercial assays was used to analyze single nucleotide polymorphisms for HOTAIR (rs920778), MIR155HG (rs1893650), TERC (rs10936599), miR-155 (rs767649), miR-196a2 (rs11614913), miR-146a (rs2910164) by allele discrimination on a Quant Studio 5 system (Applied Biosystems, Foster City, CA, USA). Supplementary Table S1 provides information on the gene variants examined.

### 2.4. Statistical Analysis and Bioinformatics

SPSS 20.0 software was used for statistical analysis of the obtained data. Nonparametric variables were analyzed using the χ2 test or Fisher’s test if the expected frequency was less than 5. The Kaplan–Meier method and the log-rank test were used to calculate progression-free survival (PFS). Progression-free survival was defined as the time from the date of diagnosis to the first evidence of tumor recurrence or survival status at the last follow-up. Hazard ratios (HR) with 95% confidence intervals (95% CI) were estimated using Cox hazard regression analysis. A univariate Cox proportional hazards analysis was initially performed to identify pathohistologic features and genetic variants associated with PFS. Variables found to be significant in the univariate analysis, including those with significance levels below 10%, were subsequently analyzed in the multivariate Cox proportional regression model to simultaneously assess the influence of multiple factors on PFS. The Cox model was calculated using the forward stepping technique, and variables with *p* < 0.1 were excluded. Logistic regression analysis adjusted for sex and age was used to test the association between analyzed polymorphisms and PTC risk. The strength of the association was assessed using the odds ratio (OR) and 95% CI. Genotypic, additive, recessive, dominant, and over-dominant models were used for risk assessment. All *p* values below 0.05 were considered significant.

The HaploReg v4.2 platform was assessed to predict the potential impact of the candidate polymorphisms on transcription factor binding motifs and/or enhancers [36]. TANRIC bioinformatics platform that associates the expression profiles from the compiled deep sequencing lncRNA data from The Cancer Genome Atlas (TCGA) database was utilized to explore associations of candidate lncRNAs with clinical characteristics or survival in the available global data on thyroid cancer [37].

## 3. Results

Demographic characteristics of studied patients and control cohort and genotype frequencies of the examined genetic variants are listed in Table 1. The patient cohort consisted of 67 women and 39 men. The median age of PTC patients was 42 years, ranging from 20 to 80 years. The mean tumor size was 23.22 ± 15.54 mm. Among 102 PTC patients, 30.4% had WHO stage I (31/102 patients), 58.8% had stage II (60/102 patients), and 10.8% had stage III (11/102 patients) tumors. The average follow-up period was 58 months (range 28–120 months). The recurrence rate was 13.7% (14/102 patients), and the majority of patients experienced disease recurrence within two years of surgery. The recurrence period ranged from 5 to 65 months, with a median time to recurrence of 14 months and a mean time of 15.71 months. None of the patients had distant metastases, and none died during the follow-up period.

A significant difference in genotype distribution was observed between PTC patients and the age- and sex-matched control group for the HOTAIR rs920778 and MIR155HG rs1893650 polymorphisms (*p* = 0.016 and *p* = 0.0002, respectively), Table 1. No differences in genotype frequencies between PTC patients and controls were identified for analyzed genetic variants in TERC, miR-155, miR-196a2, and miR-146a.

PTC patients with the TT variant genotype of the HOTAIR polymorphism rs920778 had a higher prevalence of lymph node metastases compared to the CC genotype (*p* = 0.042) and compared to C allele carriers (combined CC + CT) (*p* = 0.022), Table 2.

Furthermore, PTC patients with the TT variant genotype of HOTAIR rs920778 SNP had a higher prevalence of recurrences compared to the CC genotype (*p* = 0.031) and compared to C allele carriers (combined CC + CT genotypes) (*p* = 0.016), Table 2. In addition, the HOTAIR polymorphism rs920778 was associated with vascular invasion (*p* = 0.024) and calcifications (*p* = 0.045), Table 2. The association between MIR155HG rs1893650 variation and multifocality (*p* = 0.030) and between miR-196a2 rs11614913 SNP and ATA risk (*p* = 0.012) was also observed. The miR-155 rs767649 polymorphism was associated with the PTC stage (*p* = 0.035) and calcifications (*p* = 0.049). Age, sex, Hashimoto thyroiditis, ETE, and T category were not associated with the investigated SNPs.

**Figure 1:**
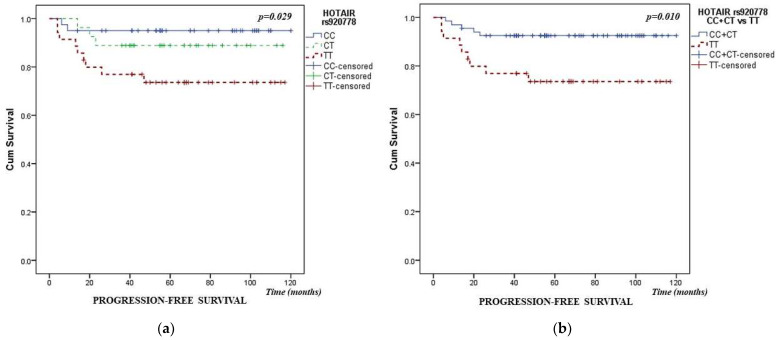
Kaplan–Meier curves of progression-free survival (PFS) of PTC patients regarding the HOTAIR rs920778 polymorphism (**a**) PFS curves for CC, CT, and TT genotypes of HOTAIR rs920778 polymorphism (**b**) combined CC and CT genotypes versus TT genotype of HOTAIR rs920778 polymorphism. *p* values were calculated according to the log-rank test.

The Kaplan–Meier method was used to estimate progression-free survival in PTC patients. A comparison of individual genotypes showed that the HOTAIR rs920778 polymorphism was associated with lower PFS, *p* = 0.029, Figure 1a. The variant TT genotype of HOTAIR rs920778 polymorphism had significantly lower PFS compared to combined CC and CT carriers, *p* = 0.010, Figure 1b. Analysis of the other individual SNPs examined revealed no association with PFS.

Relevant prognostic factors were examined in Cox logistic regression analysis as potential predictors of progression-free survival (PFS) in PTC patients, Table 3. Cox univariate logistic regression revealed ETE (hazard ratio (HR) = 4.099, *p* = 0.009), ATA risk (HR = 11.994, *p* = 0.000004), nodal metastases (HR = 2.565, *p* = 0.028), tumor stage (HR = 2.405, *p* = 0.013) and HOTAIR polymorphism rs920778 (HR = 2.467, *p* = 0.015) as significant prognostic indicators, Table 3. Variables found to be statistically significant in the univariate analysis, including those with a significance level of less than 10%, were then evaluated simultaneously in the multivariate analysis. Multivariate Cox regression analysis revealed that ATA risk (HR = 14.210, *p* = 0.000004) and HOTAIR polymorphism rs920778 (HR = 2.811, *p* = 0.010) emerged as significant prognostic factors for progression-free survival in PTC. The examined variations in the other genes had no statistically significant influence on hazard risk, Table 3.

The odds ratio adjusted for age and sex, possible confounders, revealed that HOTAIR rs920778 is associated with PTC susceptibility in the additive model, OR = 1.497, *p* = 0.021, Table 4. The variant TT genotype of HOTAIR rs920778 SNP significantly increased the risk of PTC compared to the CC genotype, OR = 2.466, *p* = 0.012, Table 4. An increased risk for the TT genotype of HOTAIR rs920778 was observed in the recessive model compared to combined C allele carriers (combined CC + CT genotype), OR = 2.512, *p* = 0.006, Table 4.

The MIR155HG rs1893650 polymorphism was significantly associated with the decreased PTC risk in the additive model (OR = 0.610, *p* = 0.029) and in the over-dominant model (OR = 0.268, *p* = 0.00007), Table 4, indicating that the heterozygote TC genotype could influence in reducing the risk of PTC. Other examined variants in the TERC, miR-146a, miR-155, and mir-196a2 genes in the studied cohort showed no correlation with cancer risk, Table 4.

Functional analysis using HaploReg v4.1, a resource for studying chromatin states and regulatory motif changes, revealed that selected candidate polymorphisms can cause enhancer histone marks as well as motif changes that can lead to allele-specific binding of transcription factors, HOTAIR rs920778, causing the motif changes for potential binding of DMRT4, DMRT5, THAP1, and MIR155HG rs1893650 causing the motif changes for potential binding of AP-2, Rad21, SMC3, Supplementary Table S1. TANRIC analysis of the global TCGA data for the expression of candidate lncRNAs in thyroid cancer revealed that HOTAIR expression is associated with the tumor stage (*p* = 0.019) and survival (*p* = 0.028). TANRIC analysis of MIR155HG expression shows a trend toward association with disease stage (*p* = 0.081), while TERC expression was not associated with clinicopathological features of thyroid cancer in TCGA data.

## 4. Discussion

Papillary thyroid carcinoma is the most common type of endocrine malignant tumor and is characterized by a favorable prognostic outcome. However, a significant increase in incidence has been observed in recent decades [1]. A number of studies have demonstrated the important role of noncoding RNAs, particularly lncRNAs and miRNAs, in the development and progression of various tumors, including PTC [5]. Noncoding RNAs play important roles in a variety of cellular processes in cancer, including proliferation, apoptosis, and metastasis [6]. Research on ncRNA gene polymorphisms is still emerging, and their associations with cancer risk, prognosis, or clinical outcomes are not yet fully elucidated. Only a limited number of studies examined the lncRNAs and miRNAs polymorphisms in PTC patients [30,31,32,33], and accumulating evidence on other types of cancers suggests that they have the potential to become biomarkers of disease susceptibility, diagnosis, and prognosis.

Our study demonstrated that the HOTAIR rs920778 polymorphism significantly contributes to increased PTC susceptibility. Furthermore, we observed a significant association of the HOTAIR rs920778 variant allele T with lymph node metastasis, tumor recurrence, and progression-free survival. Multivariate Cox regression analysis showed that initial ATA risk classification and the HOTAIR rs920778 polymorphism independently predicted the relapse of PTC. In addition, we discovered a novel association of the rs1893650 polymorphism in the lncRNA MIR155HG gene with the reduced PTC risk and showed that this polymorphism decreases the PTC risk under the over-dominant model, indicating that TC carriers could have a protective effect on PTC, exerting a heterozygote advantage.

Our results suggest that the TT genotype of the HOTAIR polymorphism rs920778 is a predisposing factor for PTC, which is consistent with previous findings for PTC in Chinese Han populations [33]. In addition, the HOTAIR rs920778 T allele contributes to an increased risk of head and neck cancer of esophageal origin [32], breast cancer in the Chinese [38] and Iranian populations [39], as well as gastric cancer [40] and colorectal cancer [41]. In addition, several meta-analyses showed that the HOTAIR rs920778 variant increases the overall risk of cancer [42,43].

Our results showing that carriers of the HOTAIR rs920778 TT genotype have a higher incidence of lymph node metastasis, higher rates of tumor recurrence, and lower progression-free survival are consistent with previous studies associating this polymorphism with the progression of multiple cancers. The TT genotype of HOTAIR rs920778 and variant T allele carriers are significantly associated with advanced tumor stage, lymph node metastasis, and poor survival rate in ovarian cancer [44]. The same polymorphism is associated with advanced tumor stage, tumor size, distant metastasis, and poor histological grade of breast carcinomas [45]. Furthermore, the HOTAIR rs920778 polymorphism is associated with worse progression-free survival in breast cancer patients [46] and colorectal cancer mortality [41].

In relevant research, Zhang et al. provided mechanistic insights into this polymorphism and showed that HOTAIR rs920778 is located in the enhancer region of intron 2 and that the T variant allele increases HOTAIR expression [32]. The functional relevance and oncogenic potential of the HOTAIR rs920778 polymorphism have been demonstrated both in vitro and in vivo in PTC. The genetic variant rs920778 has an allele-specific effect on expression, with subjects with the CT or TT genotype rs920778 having significantly higher HOTAIR expression in both normal and PTC tissues than subjects with the wild-type CC genotypes [33]. In addition, our HaploReg v4.1 analysis confirmed that HOTAIR rs920778 is associated with enhancer histone marks and also predicted motif changes that could potentially affect the binding of the transcription factor THAP1 (Thanatos-associated protein domain-containing, apoptosis-associated protein 1), previously linked to PTC [47]. In addition to binding motifs and enhancer changes, polymorphisms in the HOTAIR gene could potentially lead to changes in the secondary structure and conformation of this lncRNA that impact its allosteric interaction with different proteins.

The oncogenic potential of HOTAIR in a variety of tumor-associated processes lies in its unique role as an epigenetic master regulator, acting on both transcriptional and post-transcriptional regulation of target gene expression, thereby influencing the proliferation, migration, epithelial–mesenchymal transition (EMT), and invasion of cancer cells [14]. HOTAIR provides different binding sites to assemble histone modification enzymes, with a 5′ domain of HOTAIR (1–300 nt) binding PRC2, while a 3′ domain (1500 to 2146 nt) binds the LSD1. Acting as a molecular scaffold, HOTAIR bridges PRC2 and LSD1/CoREST/REST complexes and recruits them to the promoter regions of distant tumor suppressor and metastasis suppressor genes [16]. Furthermore, HOTAIR serves as a competing endogenous RNA to “sponge” microRNAs [48,49], thereby modulating the de-repression of miRNA targets and providing the complex cross-talk between upregulated HOTAIR and various microRNAs. High HOTAIR expression promotes the recruitment of macrophages and myeloid suppressor cells into the tumor microenvironment through the secretion of cytokines and/or chemokines by hepatocellular tumor cells [8,50].

In addition, HOTAIR promotes the secretion of exosomes [51], extracellular vesicles that act as paracrine effectors, and mediate cell-to-cell communication via the transport of active biomolecules such as proteins, lipids, and ncRNAs. Exosomal HOTAIR was shown to induce macrophage M2 polarization through activation of the PI3K/AKT signaling pathway, thereby promoting EMT and metastasis of laryngeal carcinoma cells [52].

Compelling evidence suggests that aberrantly expressed HOTAIR plays a role in thyroid cancer progression. Several recent genome-wide analysis (GWAS), microarray, and ddPCR studies show that aberrant HOTAIR expression is associated with the clinicopathological features of PTC, such as lymph node metastasis, tumor stage, and response to therapy [13,53]. High HOTAIR expression is associated with poor survival of PTC patients in clinical samples and TCGA data [54,55], indicating the potential oncogenic role of this lncRNA in PTC and an association with cancer progression. HOTAIR expression is significantly higher in the serum of PTC patients with lymph node metastases than in metastasis-negative patients [15]. In combination with galactin-3, high serum expression of HOTAIR indicates more advanced PTC [11]. Furthermore, HOTAIR is overexpressed in PTC tissues compared to non-cancerous thyroid samples [33,53] and could distinguish benign thyroid lesions from PTC [10,11,12,13]. Overexpression of HOTAIR induces the proliferation, invasion, and migration of PTC cells via the miR-488 sponging [49]. HOTAIR promotes proliferation and inhibits apoptosis of thyroid cancer cell lines (HTh-7, CAL-62, BCPAP) by silencing the expression of protein phosphatase methylesterase 1 (PPME1) by miR-761 sponging [48]. In vitro experiments with PTC cells revealed that HOTAIR promotes EMT, which is crucial for migration, invasion, and acquisition of stemness in PTC tumor cells through modulation of the Wnt/Catenin signaling pathway [15]. Overexpression of HOTAIR affects survival in vivo in the xenograft tumor model with HTh7 thyroid cancer cells in nude mice [48]. These findings indicate the oncogenic role of HOTAIR in regulating hallmark features of thyroid cancer and key signaling pathways essential for neoplastic transformation.

To date, only several studies have reported the association of MIR155HG polymorphisms with cancer risk and prognosis. To the best of our knowledge, our study provides the first report that the MIR155HG polymorphism rs1893650 could have a protective effect on thyroid cancer. The MIR155HG rs1893650 was previously found to significantly reduce liver cancer risk in the recessive model [56] but also to increase the risk of gastric cancer [57] and colorectal cancer [58]. Nevertheless, previous studies were all conducted on the Han Chinese population, which has significant population differentiation and genetic differences compared to our Caucasian Serbian population.

The MIR155HG rs1893650 polymorphism is located in the intron of the MIR155HG locus, and the functional relevance of this polymorphism has not yet been determined. Due to the dual function of the MIR155HG locus, which encodes both miR-155 and the lncRNA MIR155HG, the genetic variation of the MIR155HG gene might potentially have an impact on the transcription of both ncRNAs. Genetic variations in the binding motifs of both HOTAIR and MIR155HG that function as molecular scaffolds can hinder their interaction with proteins that alter chromatin state or control transcription. To explore this, we evaluated HaploReg v4.1 [36], which predicted that MIR155HG rs1893650 is associated with enhancer histone marks and motif changes that could potentially affect the binding of transcription factors and proteins previously associated with PTC, such as AP-2 [59] and Rad21 [60]. While the underlying role of MIR155HG and its expression in PTC remain elusive, recent studies have shown that MIR155HG acts as an oncogene in several cancer types [19,20,21]. TCGA and GTEx gene expression data showed that MIR155HG is overexpressed in a variety of tumors and that high expression of MIR155HG is strongly associated with better survival in cholangiocarcinoma, lung adenocarcinoma, and cutaneous melanoma, while its overexpression is associated with worse survival in glioblastoma, renal clear cell carcinoma, glioma, and uveal melanoma [19]. MIR155HG is associated with poor prognosis and tumor progression in glioma [21] and pancreatic cancer [61]. Lower MIR155HG expression in colorectal cancer tumor tissue correlated with both shorter overall survival and disease-free survival of patients [62]. Furthermore, MIR155HG is overexpressed in tissue samples of laryngeal cancer, and its high expression is associated with lymph node metastasis and advanced tumor stage [20].

The transcription of MIR155HG is regulated by multiple transcription factors, including NF-κB. MIR155HG is a direct target gene of NF-κB, whose inducers have been shown to increase miR-155 expression in hematopoietic cancers [22]. Overexpression of MIR155HG promotes gastric cancer proliferation, migration, and chemoresistance via NF-B and STAT3 (signal transducer and activator of transcription 3) signaling pathways [63]. Furthermore, MIR155HG and miR-155-5p are upregulated by transforming growth factor β (TGF-β) induction, which subsequently promotes the progression and EMT of laryngeal squamous cell carcinoma [20].

Possible molecular mechanisms of MIR155HG action that could be affected by genetic polymorphisms include microRNA sponging. MIR155HG is involved in the downregulation of miR-155 through its sponging, which affects proliferation, EMT, and invasion of glioma [21] and ovarian cancer [64]. MIR155HG induces proliferation, migration, and invasion in lung carcinoma by downregulating TP53INP1 via miR-155 [65]. Knockdown of MIR155HG significantly inhibits growth and promotes apoptosis in pancreatic cancer cells through miR-802 sponging [61].

MIR155HG plays a crucial role in regulating innate immunity, far beyond the processing of miR-155. Knockout mouse models lacking most of the MIR155HG sequences and another lacking the core miRNA-155 sequence showed that MIR155HG and miR-155 have distinct functions in immunity, with MIR155HG acting through IFN production and miR-155 acting through the regulation of the STAT1 signaling pathway [66]. While MIR155HG is a direct target of NF-κB that promotes its transcription [22], in a positive feedback mechanism, the MIR155HG/miR-155 axis also regulates NF-κB activity by interfering with its upstream elements or related signaling molecules and induces inflammation and the release of proinflammatory cytokines [67].

MIR155HG has a pivotal role in the regulation of M1/M2 macrophage balance and macrophage infiltration. MIR155HG promotes M2 macrophage polarization and tumor progression and enhances oxaliplatin resistance in colorectal cancer cells via the miR-650/ANXA2 axis [68]. Conversely, cytokines in chronic obstructive pulmonary disease overexpression of MIR155HG promotes polarization of M1 macrophages and release of proinflammatory, while the knockdown of MIR155HG inhibits polarization of M1 macrophages and increases the polarization of M2 macrophages [69], suggesting a differential role of MIR155HG in chronic inflammation and antitumor immune response. In addition, the expression of MIR155HG significantly correlates with the amount of infiltrating immune cells and levels of immune checkpoint molecules such as programmed cell death protein 1 (PD-1), PD-1 ligand 1 (PD-L1), and cytotoxic T lymphocyte-associated antigen 4 (CTLA4) in multiple cancers [19]. MIR155HG could affect immune checkpoint molecules via mir-155, which suppresses PD-L1, disrupts the PD-L1/PD-1 axis, and maintains T cell antitumor responses, potentially influencing resistance to cancer immunotherapy [26]. It was demonstrated that lipopolysaccharide (LPS) facilitates immune escape in hepatocellular carcinoma cells via epigenetic modification of MIR155HG to induce PD-L1 expression [70]. Therefore, variants in HOTAIR and MIR155HG genes could influence their roles in regulating M1/M2 macrophage balance, as well as immune cell infiltration in the tumor microenvironment and expression of immune checkpoint molecules.

To our knowledge, polymorphisms in the miR-155 gene have not been previously investigated in PTC. Our results demonstrated an association of miR-155 rs767649 polymorphism with the PTC stage and suggested a potential oncogenic role of miR-155 in PTC. Our findings are in line with previous studies associating the miR-155 rs767649 polymorphism with increased susceptibility and poor prognosis in lung cancer [71] and hepatocellular carcinoma [72]. The rs767649 polymorphism has a functional impact, and the T allele contributes to higher expression of miR-155 in both hepatocellular cancer and non-tumor tissue [72]. However, our results could be an ascertainment bias caused by either a small sample size and/or low frequency of the miR-155 rs767649 variant allele.

Our results did not show a potential association between miR-146a and miR-196a polymorphisms with PTC risk or clinicopathological features. Several previous studies indicated that the variant allele of rs2910164 miRNA-146a gene polymorphism is significantly associated with PTC risk [30,31]. However, in line with our results, two meta-analyses revealed that the miR-146a rs2910164 genetic variant was not associated with susceptibility to PTC in pooled analysis and/or subgroup results for Caucasians or Asians [30,73].

Several limitations of our study should be addressed. First, a limited number of subjects were included in the current study. Furthermore, recruiting controls from healthy individuals attending routine annual examinations rather than from the general population may lead to sampling biases, as our control group may not accurately represent the entire Serbian population. Finally, our study examined selected candidate polymorphisms in ncRNA genes and other variants that may influence PTC susceptibility and prognosis. Additionally, findings from a single Caucasian Serbian population may limit the generalizability of results to other populations. Due to differences in genetic backgrounds, the role of these polymorphisms and other variants in ncRNA genes in different ethnic groups and populations remains to be elucidated.

## 5. Conclusions

Our results demonstrate that the HOTAIR rs920778 polymorphism significantly contributes to increased PTC susceptibility and is associated with lymph node metastasis, tumor recurrence, and progression-free survival. Multivariate Cox regression revealed that the ATA risk stratification system and the HOTAIR rs920778 polymorphism exert a strong prognostic impact on PTC. In addition, for the first time, the present results suggest a novel association of the MIR155HG rs1893650 polymorphism with the reduced PTC risk under the over-dominant model, indicating a protective effect of heterozygote carriers. Our study presents novel data on the genetic regulation of PTC, linking polymorphisms in the lncRNA genes HOTAIR and MIR155HG to PTC risk and prognosis.

Further larger and multiethnic studies are required to elucidate the full impact of the HOTAIR and MIR155HG polymorphisms as potential new biomarkers for risk assessment, early detection, and prognosis in PTC. New biomarkers are also necessary to define personalized approaches in the development of targeted therapeutics for aggressive thyroid cancers and to enable more effective stratification of PTC patients for surgical management, as prophylactic lymph node dissection in PTC patients can lead to severe complications [74]. Furthermore, polymorphisms and/or expression of noncoding RNAs, such as HOTAIR, MIR155HG, and miR-155, could potentially be future surrogate markers to predict the efficacy of immune checkpoint blockade therapy.

## Figures and Tables

**Table 1:** Demographic characteristics and gene variant prevalence in papillary thyroid patients (PTC) and controls.

Variables		Controls		PTC Cases		*p*
		N = 106	%	N = 102	%	
Sex	Male	39	36.79	31	30.39	0.379
	Female	67	63.21	71	69.61	
Age	55<	75	70.75	67	65.69	0.459
	≥55	31	29.25	35	34.31	
HOTAIR rs920778	CC	51	48.11	40	39.22	**0.016**
	CT	37	34.91	27	26.47	
	TT	18	16.98	35	34.31	
MIR155HG rs1893650	TT	53	50.00	75	73.53	**0.0002**
	TC	46	43.40	17	16.67	
	CC	7	6.60	10	9.80	
TERC rs10936599	CC	65	61.32	68	66.67	0.146
	CT	33	31.13	21	20.59	
	TT	8	7.55	13	12.75	
miR-155 rs767649	TT	95	89.62	86	84.31	0.255
	TA	11	10.38	16	15.69	
	AA	0	0	0	0	
miR-196a2 rs11614913	CC	55	51.89	56	54.90	0.149
	CT	41	38.68	29	28.43	
	TT	10	9.43	17	16.67	
miR-146a rs2910164	GG	72	67.92	72	70.59	0.473
	GC	27	25.47	20	19.61	
	CC	7	6.60	10	9.80	

PTC—papillary thyroid cancer; N—total number of patients/controls; *p* < 0.05 are presented in bold.

**Table 2:** Association of analyzed gene variants with clinicopathological variables of PTC patients.

Variables		N	HOTAIR rs920778 wt/ht/mt	MIR155HG rs1893650 wt/ht/mt	TERC rs10936599 wt/ht/mt	miR-155 rs767649 wt/ht	miR-196a2 rs11614913 wt/ht/mt	miR-146a rs2910164 wt/ht/mt
Age	<55	67	22/21/24	52/9/6	46/11/10	56/11	38/20/9	46/15/6
	≥55	35	18/6/11	23/8/4	22/10/3	30/5	18/9/8	26/5/4
	*p*/*p* *		0.142/0.414	0.404/0.733	0.287/0.534	1	0.477/0.175	0.602/0.733
Sex	Male	31	12/8/11	22/6/3	19/9/3	26/5	14/10/7	23/4/4
	Female	71	28/19/24	53/11/7	49/12/10	60/11	42/19/10	49/16/6
	*p*/*p* *		0.986/1	0.890/1	0.356/0.749	1	0.380/0.386	0.463/0.487
Multifocality	Absent	23	8/5/10	19/0/4	18/2/3	20/3	17/5/1	16/5/2
	Present	79	32/22/25	56/17/6	50/19/10	66/13	39/24/16	56/15/8
	*p*/*p* *		0.587/0.621	**0.030**/0.227	0.267/1	1	0.077/0.110	0.946/1
ETE	Absent	84	33/23/28	61/15/6	52/19/13	69/15	45/23/16	60/16/8
	Present	18	7/4/7	14/2/2	16/2/0	17/1	11/6/1	12/4/2
	*p*/*p* *		0.868/0.785	0.780/1	0.069/0.117	0.292	0.376/0.294	0.922/1
Vascular invasion	Absent	48	22/16/10	35/8/5	32/10/6	40/8	25/18/5	33/11/4
	Present	54	18/11/25	40/9/5	36/11/7	46/8	31/11/12	39/9/6
	*p*/*p* *		**0.024/0.012**	0.981/1	0.997/1	0.797	0.087/0.110	0.687/0.746
Calcifications	Absent	72	31/14/27	52/11/9	46/16/10	64/8	41/21/10	51/15/6
	Present	30	9/13/8	23/6/1	22/5/3	22/8	15/8/7	21/5/4
	*p*/*p* *		**0.045**/0.363	0.340/0.274	0.653/0.592	**0.049**	0.505/0.244	0.694/0.475
ATA risk	Low	7	2/2/3	6/0/1	6/1/0	6/1	3/4/0	5/1/1
	Intermediate	81	34/22/25	58/16//7	51/17/13	68/13	47/17/17	57/16/8
	High	14	4/3/7	11/1/2	11/3/0	12/2	6/8/0	10/3/1
	*p*/*p* *		0.670/0.336	0.531/0.741	0.359/0.145	0.981	**0.012**/0.071	0.985/0.873
T	T1	43	17/12/14	30/9/4	32/6/5	36/7	28/8/7	33/8/2
	T2	35	17/8/10	27/4/4	17/11/7	28/7	15/13/7	22/9/4
	T3	24	6/7/11	18/4/2	19/4/1	21/3	13/8/3	17/3/4
	*p*/*p* *		0.464/0.371	0.852/0.916	0.071/0.193	0.295	0.305/0.747	0.374/0.263
N	Absent	45	23/12/10	30/9/6	32/7/6	41/4	27/11/7	28/10/7
	Present	57	17/15/25	45/8/4	36/14/7	45/12	29/18/10	44/10/3
	*p*/*p* *		**0.042/0.022**	0.354/0.330	0.535/0.874	0.093	0.640/0.789	0.150/0.102
Stage	I	31	15/8/8	22/6/3	20/6/5	29/2	17/11/3	22/7/2
	II	60	23/16/21	43/10/7	38/14/8	46/14	34/14/12	42/10/8
	III	11	2/3/6	10/1/0	10/1/0	11/0	5/4/2	8/3/0
	*p*/*p* *		0.420/0.223	0.679/0.489	0.443/0.378	**0.035**	0.580/0.452	0.568/0.296
Recurrence	Absent	88	38/24/26	66/15/7	58/17/13	75/13	51/22/15	62/16/10
	Present	14	2/3/9	9/2/3	10/4/0	11/3	5/7/2	10/4/0
	*p*/*p* *		**0.031/0.016**	0.289/0.138	0.269/0.206	0.457	0.150/1	0.323/0.350

* *p* values for combined wild-type (wt) and heterozygote (ht) genotypes vs. mutated (mt) homozygote genotypes.

**Table 3:** Analysis of different prognostic factors in relation to progression-free survival (*PFS*), according to Cox proportional hazards regression analysis.

COX Regression Analysis	Variables	Progression-Free Survival		
		HR	(95% CI)	*p*
Univariate Analysis	Age (55 years)	1.068	(0.357–3.188)	0.907
	Sex	0.412	(0.144–1.175)	0.097
	Hashimoto thyroiditis	0.466	(0.146–1.486)	0.197
	Multifocality	3.719	(0.486–28.427)	0.206
	ETE	4.099	(1.420–11.831)	**0.009**
	Vascular invasion	1.768	(0.592–5.279)	0.308
	ATA risk	11.994	(4.176–34.448)	**0.000004**
	T	1.482	(0.799–2.751)	0.212
	Nodal metastases	2.565	(1.107–5.941)	**0.028**
	Stage	2.405	(1.206–4.798)	**0.013**
	Calcifications	0.368	(0.082–1.644)	0.190
	HOTAIR rs920778	2.467	(1.195–5.094)	**0.015**
	TERC rs10936599	0.649	(0.263–1.601)	0.348
	MI155HG rs1893650	1.607	(0.794–3.253)	0.187
	miR-155 rs767649	1.474	(0.411–5.289)	0.552
	miR-196a2 rs11614913	1.286	(0.673–2.458)	0.446
	miR-146a rs2910164	0.755	(0.305–1.873)	0.544
Multivariate Analysis	ATA risk	14.210	(4.589–43.999)	**0.000004**
	HOTAIR rs920778	2.811	(1.275–6.197)	**0.010**

HR indicates a hazard ratio; CI, confidence interval; *p* < 0.05 are presented in bold.

**Table 4:** Association of analyzed gene polymorphisms and PTC risk.

Gene/SNP	Genotype	Controls		PTC Cases		Age and Sex Adjusted OR, (95% CI)	*p*
		N = 106	%	N = 102	%		
HOTAIR rs920778	CC	51	48.11	40	39.22	1	Reference
	CT	37	34.91	27	26.47	0.956 (0.499–1.832)	0.892
	TT	18	16.98	35	34.31	2.466 (1.219–4.990)	**0.012**
	Additive model					1.497 (1.063–2.110)	**0.021**
	Recessive model-mt vs. wt + ht (Ref.)					2.512 (1.306–4.829)	**0.006**
	Dominant model-wt vs. ht + mt (Ref.)					0.684 (0.393–1.190)	0.179
	Over-dominant model-ht vs. wt + mt (Ref.)					0.693 (0.380–1.261)	0.230
MIR155HG rs1893650	TT	53	50.00	75	73.53	1	Reference
	TC	46	43.40	17	16.67	0.268 (0.139–0.520)	**0.00009**
	CC	7	6.60	10	9.80	1.013 (0.361–2.846)	0.980
	Additive model					0.610 (0.392–0.951)	**0.029**
	Recessive model-mt vs. wt + ht (Ref.)					1.841 (0.639–5.304)	0.258
	Dominant model-wt vs. ht + mt (Ref.)					1.921 (0.665–5.546)	0.228
	Over-dominant model-ht vs. wt + mt (Ref.)					0.268 (0.140–0.513)	**0.00007**
TERC rs10936599	CC	65	61.32	68	66.67	1	Reference
	CT	33	31.13	21	20.59	0.586 (0.304–1.129)	0.110
	TT	8	7.55	13	12.75	1.666 (0.636–4.362)	0.299
	Additive model					1.005 (0.670–1.510)	0.979
	Recessive model-mt vs. wt + ht (Ref.)					1.345 (0.844–2.142)	0.212
	Dominant model-wt vs. ht + mt (Ref.)					1.234 (0.693–2.197)	0.476
	Over-dominant model-ht vs. wt + mt (Ref.)					0.584 (0.308–1.108)	0.100
miR-155 rs767649	TT	95	89.62	86	84.31	1	Reference
	TA	11	10.38	16	15.69	1.655 (0.718–3.813)	0.237
	Additive model					1.578 (0.688–3.618)	0.282
miR-196a2 rs11614913	CC	55	51.89	56	54.90	1	Reference
	CT	41	38.68	29	28.43	0.760 (0.411–1.405)	0.381
	TT	10	9.43	17	16.67	1.663 (0.687–4.025)	0.260
	Additive model					1.115 (0.756–1.645)	0.582
	Recessive model-mt vs. wt + ht (Ref.)					1.404 (0.923–2.137)	0.113
	Dominant model-wt vs. ht + mt (Ref.)					1.845 (0.785–4.334)	0.160
	Over-dominant model-ht vs. wt + mt (Ref.)					0.646 (0.359–1.160)	0.143
miR-146a rs2910164	GG	72	67.92	72	70.59	1	Reference
	GC	27	25.47	20	19.61	0.817 (0.415–1.609)	0.558
	CC	7	6.60	10	9.80	1.329 (0.471–3.747)	0.591
	Additive model					1.001 (0.650–1.540)	0.997
	Recessive model-mt vs. wt + ht (Ref.)					1.178 (0.649–2.137)	0.591
	Dominant model-wt vs. ht + mt (Ref.)					1.391 (0.498–3.881)	0.529
	Over-dominant model-ht vs. wt + mt (Ref.)					0.664 (0.340–1.297)	0.231

## Data Availability

The data presented in this study are available on request from the corresponding author. The data are not publicly available due to privacy and ethical restrictions.

## Supplementary Materials

The following supporting information can be downloaded at: https://www.mdpi.com/article/10.3390/cancers16030485/s1, Table S1: Characteristics of genotyped polymorphisms and HaploReg v4.2 prediction.
