# Supplementary material for: Association of HOTAIR, MIR155HG, TERC, miR-155, -196a2, and -146a Genes Polymorphisms with Papillary Thyroid Cancer Susceptibility and Prognosis
Source: Cancers (Basel). 2024 Jan 23;16(3):485. doi: 10.3390/cancers16030485 (PMC10854783; doi:10.3390/cancers16030485)
Supplement: Supplementary file 1 [file cancers-16-00485-s001.zip › cancers-2675752-supplementary.pdf]

Supplement Table S1. Characteristics of genotyped polymorphisms and HaploReg v4.2 prediction.

Gene	Gene Locus	rs number	SNP Change	Region	HaploReg v4.2 prediction	
					**Enhancer histone marks**	**Motifs changed**
**HOTAIR**	12q13.13	***rs920778***	C/T	intron enhancer	+	DMRT4, DMRT5, THAP1
**MIR155HG**	21q21.3	***rs1893650***	T/C	intron	+	AP-2, Rad21, SMC3
**TERC**	3q26.2	***rs10936599***	C/T	intron	-	Eomes, NRSF, Zfp691
**miR-155**	21q21.3	***rs767649***	A/T	promoter	+	Irf, Mrg1, Hoxa9, PRDM1
**miR-196a2**	12q13.13	***rs11614913***	C/T	3’ UTR	+	HMG-IY
**miR-146a**	5q33.3	***rs2910164***	C/G	promoter	+	-
